# Effectiveness and safety of moxibustion for primary insomnia: a systematic review and meta-analysis

**DOI:** 10.1186/s12906-016-1179-9

**Published:** 2016-07-13

**Authors:** Yu-Jiao Sun, Jia-Min Yuan, Zhi-Min Yang

**Affiliations:** Guangzhou University of Chinese Medicine, No.12, Jichang Road, Bai Yun District, Guangzhou, Guangdong 510405 China; Guangdong Hospital of Traditional Chinese Medicine, No.111, Dade Road, Yue Xiu District, Guangzhou, Guangdong 510120 China

**Keywords:** Moxibustion, Insomnia, Systematic Review

## Abstract

**Background:**

Primary insomnia is a widespread and refractory disease. Moxibustion therapy for insomnia shows some advantages compared with conventional therapies. This systematic review and meta-analysis of randomized controlled trials (RCTs) was conducted to evaluate the effectiveness and safety of moxibustion therapy for insomnia.

**Methods:**

We conducted a comprehensive literature review of the CENTRAL, PubMed, EMBASE, Web of science, CNKI, VIP, and Wanfang Data databases from their inception to July 2015 for RCTs that compared moxibustion with western medications, oral Chinese medicine, or other methods of traditional Chinese medicine (TCM) in patients with primary insomnia. The primary outcome measure was effective rate and secondary outcome measure was adverse events. Data collection and analysis included risk of bias evaluation, meta-analysis, sensitivity analysis, publication bias and adverse events analysis according to corresponding criteria.

**Results:**

The study included 22 RCTs (1,971 patients). The quality of the studies was low. The overall meta-analysis demonstrated that moxibustion was more effective for insomnia than western medications, oral Chinese medicine and other TCM therapies (RR = 1.17, 95 % CI 1.12 to 1.23, *P* < 0.00001). Subgroup analyses demonstrated that moxibustion was more effective for insomnia than western medications (RR = 1.16, 95 % CI 1.09 to 1.24, *P* < 0.00001), oral Chinese medicine (RR = 1.11, 95 % CI 1.04 to 1.18, *P* = 0.002), and other TCM therapies (RR = 1.22, 95 % CI 1.15 to 1.30, *P* < 0.00001). There were no serious adverse effects associated with moxibustion therapy for insomnia, and the rate of adverse events was low.

**Conclusion:**

It is difficult to get the conclusion regarding the effectiveness and safety of moxibustion for primary insomnia due to insufficient evidence, such as the high risk of bias in the included studies, small sample sizes, and few reports on adverse effects. Moxibustion should be considered as a novel therapeutic option for insomnia, and more rigorous clinical trials of moxibustion therapy for insomnia are needed to assess its effects.

## Background

### Description of the condition

Insomnia is a sleep disorder characterized by the inability to fall asleep, sleep loss and poor-quality sleep. Insomnia is caused by multiple physiological, psychological, and environmental factors [1–4].

Insomnia is related to the function of the cerebral cortex and results from mental or nervous tension. Insomnia is thought to be associated with a group of centrally located neurons coupled with dynamic transformation of neurotransmitters, including norepinephrine (NE) and 5-hydroxytryptamine (5-HT) [5].

In industrialized countries, insomnia is an epidemic [6–9], where an estimated 40 % of the population suffers from the disorder [10–16]. An international survey published in 2008 estimated the prevalence of insomnia at 23 % in Japan, 31 % in Western Europe, and 56 % in the United States [17].

Insomnia can lead to memory problems, depression [18–20], irritability, and an increased risk for cardio-cerebrovascular diseases [21–23], such as headache [24], hypertension [25–27], and heart failure [28]. Insomnia can decrease an individual’s health-related quality of life [29], leading to functional impairment while awake [29–31] and consequences such as automobile-related accidents [32] and absenteeism [33–37].

However, insomnia remains under-diagnosed and under-treated. An estimated 47-67 % of individuals with insomnia do not seek medical attention. Among those that do attempt to resolve their sleep problems, only 50–90 % receive treatment [17, 38].

### Description of the intervention

Western conventional medicine recommends pharmacological treatment (such as hypnotic sedative agents) and cognitive behavioral therapy (CBT) for insomnia [39].

Pharmacological agents are effective for insomnia but are only recommended for short-term relief. The long-term use of these medications is associated with adverse effects such as disturbed sleep architecture, rebound insomnia, withdrawal effects [40], damage to cerebral nerves, memory and psychomotor impairment, hypofunction, dependency, and addiction [41]. For example, benzodiazepines may cause headaches, nightmares, daytime fatigue, nausea, confusion, and falls [42]. Z-drugs can result in bizarre behaviors, dizziness, falls, and gastrointestinal upset [43].

Evidence from clinical studies supports the use of CBT for insomnia [44, 45]. However, CBT is not effective in all patients [46], and access to treatment is limited [45, 47] because qualified CBT therapists are rare [48] and expensive [49].

Consequently, insomnia sufferers require alternative treatments [50, 51]. Moxibustion is a component used in traditional Chinese medicine (TCM). Some Chinese studies by randomized controlled trials (RCTs) or clinical observations suggest that the moxibustion has the potential to be an effective and safe therapy for insomnia, such as improving sleep quality, adjusting the brain’s sleep function, improving symptoms of dreaminess, dizziness, headache, heavy head and poor memory, and promoting the periodicity from light to deep sleep [52].

### How the intervention might work

Modern medicine believes moxibustion modulates neurotransmitters to resist insomnia, thereby improving sleep quantity [53]. Experiments in rats indicate that moxibustion protects against chronic stress by acting on the hippocampal neurons to increase the amount of brain-derived neurotrophic factor as well as 5-HT and its metabolites. Holistic healthcare uses moxibustion to generate far-infrared and near infrared energy to regulate dysfunctional organs and build wellbeing [54]. Suspended moxibustion at Baihui can treat nervous system diseases by improving the blood supply to brain tissue, increasing the elasticity of blood vessels, and enhancing the excitability of related sites on the cerebral cortex [54]. In TCM, moxibustion is thought to regulate *qi* and the blood, tonifying healthy *qi* to eliminate pathogenesis by means of warming. Moxibustion applied at Bǎihuì can balance yin and yang, tonify both the heart and the spleen, dredge blood vessels, and tranquilize [54] mind [5].

### Why it is important to do this study

The effectiveness of moxibustion therapy for insomnia remains controversial; therefore, its application is limited. There are currently no published systematic reviews or meta-analyses investigating the effectiveness and safety of moxibustion therapy for insomnia.

## Objective

This systematic review and meta-analysis of randomized controlled trials (RCTs) was conducted as a rigorous evaluation of the effectiveness and safety of moxibustion therapy for insomnia.

## Methods

This systematic review and meta-analysis is reported according to the Preferred Reporting Items for Systematic Reviews and Meta-Analyses (PRISMA) guidelines [55].

### Database and search strategy

Two review authors (SYJ and YJM) independently searched the Cochrane Central Register of Controlled Trials (CENTRAL), PubMed, EMBASE, Web of Science, Chinese National Knowledge Infrastructure (CNKI), VIP information database, and Wanfang Data Information Site from their inception to July 2015. Searches were restricted to studies in the English and Chinese languages.

Medline (Pubmed) search strategy:

1. insomnia OR dyssomnia OR sleep OR sleep disorder OR sleep maintenance OR somnipathy (in full text);
2. moxibustion OR moxa (in full text);
3. clinical trial OR controlled clinical trial OR randomized controlled trial OR randomized clinical trial (in full text);
4. #1 AND #2 AND #3

A monthly e-mail alert was set up at the National Center for Biotechnology Information (NCBI) from the U.S. National Library of Medicine (NLM) to obtain updates of new publications.

### Inclusion criteria

1. RCTs of patients that were dissatisfied with their quality of sleep;
2. in which the intervention group included patients undergoing therapy with different methods of moxibustion as monotherapy or combination therapy (including grain-moxibustion, thunder-fire moxibustion, heat-sensitive moxibustion), and the control group included patients undergoing therapy with western medications, or oral Chinese medicine, or other TCM therapies (including acupoint massage, point-application, head-needle acupuncture, auricular-plaster therapy, and acupuncture);
3. The primary outcome measure was the clinical effective rate. It was a dichotomous outcome and the overall effectiveness of moxibustion therapy as a subjective assessment, which was defined as the proportion of participants who got improved in sleep quality and was based on response evaluation criteria used in the treatment of insomnia with TCM. What’s more, it was reported by trial participants themselves.
  For example, clinical therapeutic effect criteria was categorized as cure, markedly effective, effective, or ineffective. according to the Guideline for Clinical Trials of New Patent Chinese medicines (GCTNPCM) evaluation standards, which define: (1) clinical cure as sleep time to restore normal sleep time OR a nighttime sleep duration of < 6 h, deep sleep, and full of energy after waking up; (2) markedly effective as significant improvement of insomnia, sleep time increased < 3 h compared to previous sleep time and an increase in the depth of sleep; (3) effective as amelioration in symptoms as sleep time increased < 3 h compared with the previous sleep time; and (4) ineffective as no significant improvement of insomnia OR deteriorated after treatment [39, 56]. Then the patients of “cure, markedly effective, effective” were taken as people who got improved in sleep quality and the patients of “ineffective” were taken as people who got unimproved in sleep quality. The total number of “cure, markedly effective, effective” were used to calculate effective rate.
  Other assessment criteria of clinical therapeutic effect with comparable definitions were also considered [39].
4. The secondary outcome measure was adverse events associated with the use of moxibustion therapy for insomnia. It was reported in the articles or measured by validated scales, e.g., Health Survey Questionnaire, Treatment Emergent Symptom Scale (TESS) et al.
5. Only English and Chinese as language selection

### Exclusion criteria

1. studies that were not RCTs;
2. patients diagnosed with primary insomnia resulting from another physiological or psychological disease;
3. trials in special patient populations such as menopausal women;
4. trials in which moxibustion as combination therapy was not the only intervention to differ between the treatment and control group;
5. studies reporting fraudulent data or with insufficient data;
6. duplicate studies.

### Study selection

Two review authors (SYJ, YJM) independently examined titles and abstracts to select eligible RCTs. When datasets overlapped or were duplicated, only the most recent information was included. Then the full text of potentially relevant studies was retrieved. Two author reviewers (SYJ and YJM) independently examined the full text records to determine which studies met the inclusion criteria. Disagreements about the study selection were resolved by discussion with a third review author (YZM) and consensus.

### Data extraction and management

Two review authors (SYJ and YJM) independently extracted the data from eligible RCTs including details on the study population, interventions, and outcome measures. Disagreements about data extraction were resolved through discussion with a third review author (YZM) and consensus.

### Assessment of quality of evidence in included studies

The methodological quality of RCTs was assessed independently using the Cochrane Handbook for Systematic Reviews of Interventions [57] from 7 parts, including random sequence generation, allocation concealment, blinding of participants and personnel, blinding of outcome assessments, incomplete outcome data, selective reporting, and other bias.

Two review authors (SYJ, YJM) independently evaluated the methodological quality of the included articles. Disagreements about the assessment of quality of evidence in included studies were resolved through discussion with a third review author (YZM) and consensus.

RCTs with fraudulent data of low quality were not included in the meta-analysis.

## Data analysis

Statistical analyses were performed using Review Manager [Computer program] Version 5.3. (RevMan5.3, Copenhagen: The Nordic Cochrane Centre, The Cochrane Collaboration, 2014). Risk ratios (RRs) with 95 % CIs were calculated for dichotomous variables.

A random-effects model was used to pool the studies with significant heterogeneity, as determined by the inconsistency index (*I*^*2*^ ≥ 30 %). A fixed effect model was used to pool the studies in the absence of substantial heterogeneity (*I*^*2*^ < 30 %).

Sensitivity analyses were conducted to explore the impact of confounding factors.

Publication bias was comprehensively assessed using funnel plot by RevMan v5.3. and Begg’s rank correlation test of asymmetry by stata.13.0. Publication bias was thought to be insignificant at P > 0.05 [58].

## Results

### Trial identification

**Fig. 1:**
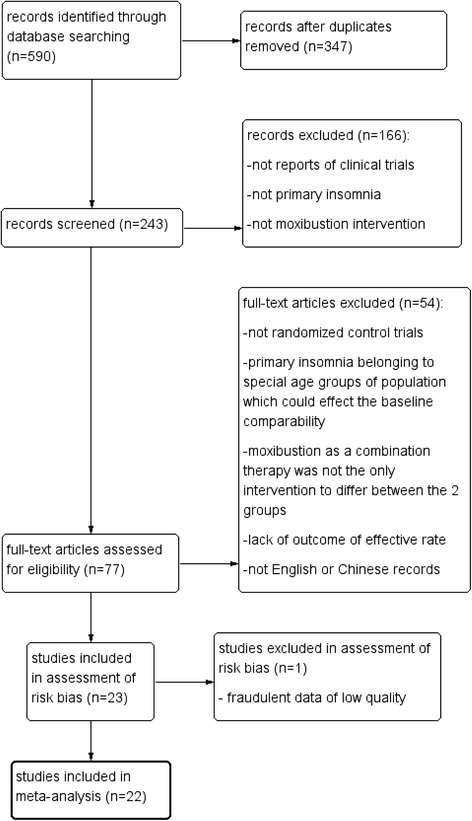
Flow chart of literature search. Following the search strategy, 590 articles were identified from medical literature databases, and 77 required further assessment. Finally, 22 articles were included in this review

The searches identified 590 articles. Titles and abstracts were screened, and 77 RCTs were considered potentially eligible for inclusion. After analyzing the full-text articles and a risk of bias assessment, 55 RCTs were excluded. Twenty-two RCTs were found eligible based on our inclusion criteria (Fig. 1).

### Characteristics of included studies

The characteristics of the 22 included trials (*n* = 1,971) were summarized in Table 1. Five trials [54, 59–62] compared moxibustion with western medications (*n* = 477; moxibustion: *n* = 241, control: *n* = 236) (Table 1a), six trials [63–68] compared moxibustion with oral Chinese medicine (*n* = 533; moxibustion: *n* = 267, control: *n* = 266) (Table 1b), and eleven trials [5, 69–78] compared moxibustion with other TCM therapies (*n* = 961; moxibustion: *n* = 491, control: *n* = 470) (Table 1c).

**Table 1:** Characteristics of trials included in the meta-analysis

a. Moxibustion vs. Western medications						
Included trials	Eligibility criteria	Interventions and treatment duration		Sample and characteristics (male female, age, disease duration)		Outcome Criteria (effective rate Criteria)
		Trial	Control	Trial	Control	
Wu 2014 [59]	CD&TETCMD&S	Moxibustion Duration:30d	Estazolam Duration:30d	91; AGE: 20-75; Disease duration:3 m-30y	91; AGE: 20-75; Disease duration:3 m-30y;	Unclear
Wong 2014 [62]	CCMD-3	Moxibustion Duration:20d	Estazolam Duration:20d	40 (M: 18, F: 22); AGE: 18-63 (mean: 38); Disease duration: 1.5 m-4y	40 (M: 16; F: 24); AGE: 18-66 (mean: 39); Disease duration:1.2 m-6y	GCTNPCM
Yu 2012 [60]	-CCMD-3 -ICD10	Moxibustion Duration:21d	Estazolam Duration: 14 d	30; AGE: 13-65; Disease duration: 1 m-8 y	30; AGE: 13-65; Disease duration:1 m-8y;	GCTNPCM
Ju 2009 [54]	Sleep efficiency computation	Moxibustion Duration:10d	Estazolam Duration: 10 d	40 (M: 18, F: 22); AGE: 25-75; Disease duration: 0.5y-5 y	35 (M: 14 F: 21); AGE: 25-75; Disease duration:0.5y-5y	Sleep efficiency computation
Yuan 2007 [61]	-CCMD-3 -ICD10	Moxibustion Duration:20d	-Diazepam -Oryzanol --V_B1_ Duration: 20 d	40; AGE 14-65; Disease duration: 1 m-10 y;	40; AGE 14-65; Disease duration:1 m-10 y;	GCTNPCM
b. Moxibustion vs. oral Chinese medicine						
Included trials	Eligibility criteria	Interventions and treatment duration		Sample and characteristics (male female, age, disease duration)		Outcome Criteria (effective rate Criteria)
		Trial	Control	Trial	Control	
Jiao 2015 [65]	-CCMD-3 -CDT&ETCMD&S	-Moxibustion -SanhuangAnshen Decoction Duration: 30d	SanhuangAnshen Decoction Duration: 30d	80 (M: 37, F: 43); AGE: (mean: 67.8); Disease duration: -;	79 (M: -, F: -); AGE: (mean: 65.1); Disease duration: -;	CDT&ETCMD&S
Liu 2015 [66]	-CCMD-3 -GCTNPCM	Grain-moxibustion Duration: 14 d	Renshenguipi pill Duration: 14 d	38; AGE: 18-70; Disease duration: -;	38; AGE: 18-70; Disease duration: -;	GCTNPCM
Zhang 2014 [68]	-CCMD-3	-Moxibustion -Huatanjieyu decoction Duration: 10 d	Huatanjieyu decoction Duration: 7 d	59; AGE: 34-65; Disease duration: -;	59; AGE: 34-65; Disease duration: -;	Unclear
He 2014 [63]	-ICD-10- -GCTNPCM	-Moxibustion -Tianwangbuxin decoction Duration: 32 d	Tianwangbuxin decoction Duration: 28 d	30 (M: 12, F: 18); AGE:30-60 (mean:45.3 ± 4. 4); Disease duration: -;	30 (M: 13, F: 17); AGE:31-60 (mean:46.2 ± 5.1); Disease duration: -;	GCTNPCM
Wu 2010 [67]	-CCMD-3 -CDT&ETCMD&S	-Heat-sensitive Moxibustion –AnshenBunao decoction Duration: 22 d	AnshenBunao decoction Duration: 22 d	30 (M: 13, F: 17); AGE:33-74 (mean:46.8); Disease duration: 1 w-2.7 y	30 (M: 11, F: 19); AGE:35-75 (mean:45.7); Disease duration: 1w-2.5 y	GCTNPCM
Hu 2007 [64]	PSQI	Moxibustion Duration: 30 d	AnshenBunao Ye Duration: 30 d	30 (M: 12, F: 18); AGE: 19-65; Disease duration: 24 d-16 y	30 (M: 14, F: 16); AGE: 18-67; Disease duration: 20 d-14 y	PSQI
c. Moxibustion vs. other TCM therapies						
Included trials	Eligibility criteria	Interventions and treatment duration		Sample and characteristics (male female, age, disease duration)		Outcome Criteria (effective rate criteria)
		Trial	Control	Trial	Control	
Xie 2015 [77]	CCMD-3	-Moxibustion -Head-acupoint massage Duration: 28 d	Head-acupoint massage Duration: 28 d	30 (M: 17, F: 13); AGE: (mean: 43.3 ± 13.8 y); Disease duration: (mean: 11.5 ± 5.3 m);	30 (M: 14, F: 16); AGE: (mean: 42.7 ± 12.4 y); Disease duration: (mean: 10.8 ± 4.7 m);	GCTNPCM
Wang 2014 [76]	Unclear	-Moxibustion -Point-application Duration:15 d	Point-application Duration:15 d	18 (M: 10, F: 8); AGE:39-65y (mean:45.2 y); Disease duration: -	18 (M: 11, F: 7); AGE:41-69y (mean:48.1 y); Disease duration: -	Unclear
Xu 2014 [78]	CD&TETCMD&S	-Heat-sensitive Moxibustion -Head-needle acupuncture Duration:24 d	Head-needle acupuncture Duration:24 d	58 (M: -, F: -); AGE: 18-70 y; Disease duration: 12 m-60 m	54 (M: -,F: -); AGE: 18-70 y; Disease duration: 12 m-60 m	sleep efficiency by international standard
Shu 2014 [75]	GCTNPCM	-Moxibustion -Head-acupoint massage Duration:15 d	Head-acupoint massage Duration: 15 d	9 (M: 0, F: 9); AGE: 30-56; Disease duration: 2 m-7 m	9 (M: 0, F: 9); AGE: 30-56; Disease duration: 2 m-7 m	GCTNPCM
Ma 2014 [73]	CCMD-3	-Moxibustion -Auricular-plaster therapy Duration:47 d	Auricular-plaster therapy Duration:47 d	99 (M: 38, F: 61); AGE: 20-64 y (mean: 38 ± 13 y); Disease duration: 0.5 y-20y (mean: 7.48 ± 4.57 y)	96 (M: 33, F: 63); AGE: 21-62 y (mean: 37 ± 12 y); Disease duration: 0.5 y-20 y (mean: 7.13 ± 4.92 y)	unclear
Li 2014 [70]	ICD-10	-Moxibustion -Acupuncture Duration:21 d	-Acupuncture Duration:21 d	35 (M: 12, F: 23); AGE: 20-60 y (mean: 45 ± 3. 5 y); Disease duration: 1 m-18 m (mean: 5.5 ± 4.2 m)	35 (M: 8, F: 27); AGE: 25-60 (mean: 48 ± 4. 9 y); Disease duration: 1 m-18 m (mean: 5.6 ± 0.4 m)	GCTNPCM
Quan 2012 [74]	CCMD-2-R	-Moxibustion -Acupuncture Duration:43 d	Acupuncture Duration:43 d	36 (M: 15, F: 21); AGE: 19-67 y (mean:38.9 y); Disease duration: 6 m-9 y (mean: 5.3 y);	36 (M: 17, F: 19); AGE: 20-68 y (mean:40.6 y); Disease duration: 5 m-10 y (mean: 5 y);	GCTNPCM
Ao 2011 [69]	CCMD-3	-Moxibustion -Acupuncture Duration:32-33 d	Acupuncture Duration:32-33d	34 (M: 13, F: 21); AGE: (mean:40.54 ± 11.27); Disease duration: (mean: 9.7 ± 2.45 m);	33 (M: 14, F: 19); AGE: (mean:39.67 ± 11.93); Disease duration: (mean: 8.47 ± 1.69 m);	Sleep efficiency by international standard
Li 2011 [5]	CCMD-3	-Moxibustion -Acupuncture Duration:28 d	Acupuncture Duration:28 d	100 (M: 46, F: 54); AGE: (mean: 35.58 ± 9.87); Disease duration: (mean: 21.59 ± 7.87 m);	98 (M: 45, F: 53); AGE: (mean: 36.67 ± 10.93); Disease duration:(mean: 22.76 ± 8.39 m);	GCTNPCM
Chen 2010 [71]	-CCMD-3 -ICD -10	-Moxibustion -Auricular-plaster therapy Duration:22-23 d	Auricular-plaster therapy Duration:22-23d	37 (M: -,F: -); AGE:18-72 (mean:48); Disease duration: 2 m-7y	26 (M: -,F: -); AGE:18-72 (mean:48); Disease duration: 2 m-7 y	Unclear
Li 2010 [72]	-CCMD-2-R -CD&TETCMD&S	-Thunder-fire moxibustion -Acupuncture Duration:10-30 d	Acupuncture Duration:10-30d	35 (M: 15, F: 20); AGE: (mean: 35.6); Disease duration: (mean: 3.3 y);	35 (M: 16, F: 19); AGE: (mean: 33.6); Disease duration: (mean: 3.6 y);	GCTNPCM

Twenty-two trials (*n* = 1,971) conducted in China were included in this study

CCMD-2-R = Chinese classification and diagnostic criteria for mental disorders second edition-revision; CCDM-3 = Chinese classification and criteria for disorders 3^rd^ edition; CDT&ETCMD&S = Criteria of Diagnosis and Therapeutic Effects for TCM Disease and Syndrome; GCTNPCM = Guideline for Clinical Trials of New Patent Chinese Medicine; ICD-10 = International Classification of Disease, 10^th^ Version; PSQI = Pittsburgh Sleep Quality Index; M = month; Y = year; D = day; W = week

Patients included in the 22 trials were 13 [60] - 75 years [59, 67] of age (moxibustion vs. western medications: range, 13 [60] - 75 years [59]; moxibustion vs. oral Chinese medicine: range, 18 [66] - 75 years [67]; moxibustion vs. other TCM therapies: range, 18 [71, 78] - 72 years [71]). The overall duration of disease in the 22 included trials ranged from 1 week [67] to 30 years [59] (moxibustion vs. western medications: range, 1 month [60] to 30 years [59]; moxibustion vs. oral Chinese medicine: range, 1 week [67] to 16 years [64]; moxibustion vs. other TCM therapies: range, 1 month [70] to 20 years [73]).

The diagnostic criteria used in the 22 trials included (1) Chinese classification and criteria for mental disorders 3^rd^ edition (CCMD-3) [5, 60–62, 65–69, 71, 73, 77], (2) Chinese classification and diagnostic criteria for mental disorders second edition-revision (CCMD-2-R) [72, 74], (3) GCTNPCM [63, 66, 75], (4) International Classification of Disease 10^th^ Version (ICD-10) [60, 61, 63, 70, 71], (5) sleep efficiency calculation of the World Health Organization (WHO) [54], (6) Criteria of Diagnosis and Therapeutic Effects for TCM Disease and Syndrome (CDT&ETCMD&S) [59, 65, 67, 72, 78], (7) Pittsburgh Sleep Quality Index (PSQI) [64], and (8) unclear criteria [76].

The treatment duration of the treatment groups ranged from 10 [54, 68, 72] to 47 days [73] (moxibustion vs. western medications: range, 10 [54] to 30 days [59]; moxibustion vs. oral Chinese medicine: range, 10 [68] to 32 days [63]; moxibustion vs. other TCM therapies: range, 10 [72] to 47 days [73]).

In the control groups of the 22 included trials, the treatment methods included 2 western medications (Estazolam [54, 59, 60, 62], Diazepam plus Oryzanol plus V_B1_ [61]), 6 oral Chinese medicine therapies (Sanhuang Anshen decoction [65], Renshenguipi pill [66], Huatanjieyu decoction [68], Tianwangbuxin decoction [63], Anshen Bunao decoction [67], Anshen Bunao Ye [64]), and 5 other TCM therapies (head-acupoint massage [75, 77], point-application [76], head-needle acupuncture [78], auricular-plaster therapy [71, 73], and acupuncture [5, 69, 70, 72, 74]). The treatment duration of the control groups ranged from 7 [68] to 47 days [73]: (moxibustion vs. western medications: range, 10 [54] to 30 days [59]; moxibustion vs. oral Chinese medicine: range, 7 [68] to 30 days [64, 65]; moxibustion vs. other TCM therapies: range, 10 [72] to 47 days [73]).

The effectiveness of moxibustion was classified according to 5 criteria: (1) GCTNPCM [5, 60–63, 66, 67, 70, 72, 74, 75, 77], (2) CDT&ETCMD&S [65], (3) PSQI [64], (4) WHO sleep efficiency calculation [54, 69, 78], (5) unclear criteria [59, 68, 71, 73, 76].

The baseline was comparable because there were no significant differences in gender, age, or disease duration between the intervention and control groups (P > 0.05).

### Risk of bias in included studies

**Fig. 2:**
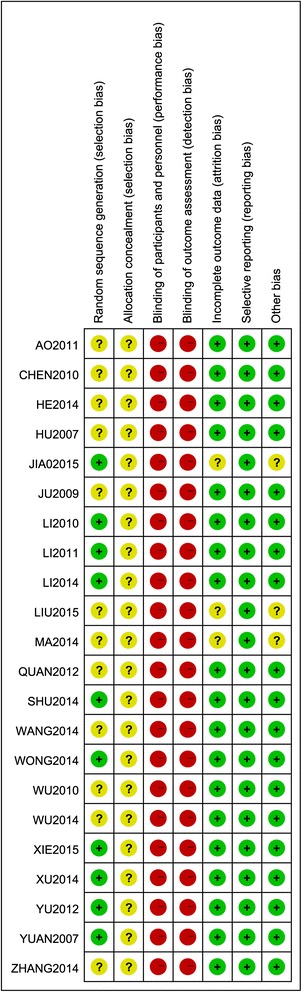
Risk of bias in included studies

The overall risk of bias in the 22 included trials was high (Fig. 2).

In 10 trials [5, 60–62, 65, 70, 72, 75, 77, 78], the risk of bias due to random sequence generation was assessed as low because a random number table was used. In 12 trials [54, 59, 63, 64, 66–69, 71, 73, 74, 76], the risk of bias due to random sequence generation was assessed as unclear due to insufficient details in the report.

In all 22 trials [5, 54, 59–78], the risk of bias due to allocation concealment was assessed as unclear due to insufficient details in the report.

In all 22 trials [5, 54, 59–78], the risk of bias due to blinding of participants and personnel was assessed as high. Blinding of participants and personnel was never possible because all the included trials adopted conventional western medications, oral Chinese herbal medicine, or other TCM interventions such as massage, or acupuncture.

In all 22 trials [5, 54, 59–78], the risk of bias due to blinding of outcome assessments was assessed as high because the primary outcome (effective rate) was reported by trial participants only according to inclusion criteria and trial participants were never possible to be blinded.

In 19 trials [5, 54, 59–64, 67–72, 74–78], the risk of bias due to incomplete outcome data was assessed as low because there were no missing data and all expected outcomes were reported. Three articles [65, 66, 73] were assessed as having unclear risk because they did not report sufficient detail to let us make sure the baseline was balanced after dropouts and did not use an intention-to-treat (ITT) analysis, though the dropouts were less than 20 % in each article.

In all 22 trials [5, 54, 59–78], the risk of bias due to selective reporting was assessed as low because all the trials reported all of the outcomes that they had specified in their methods and no additional outcomes were reported.

In 19 trials [5, 54, 59–64, 67–72, 74–78], the risk of bias due to other reasons was assessed as low because these studies appeared to be free of other sources of bias. Three articles [65, 66, 73] were assessed as being at unclear risk because there were no sufficient detail to let us make sure the baseline was balanced after dropouts.

### Effective rate of moxibustion for insomnia

The effective rate of moxibustion for insomnia was described in all 22 included trials.

**Fig. 3:**
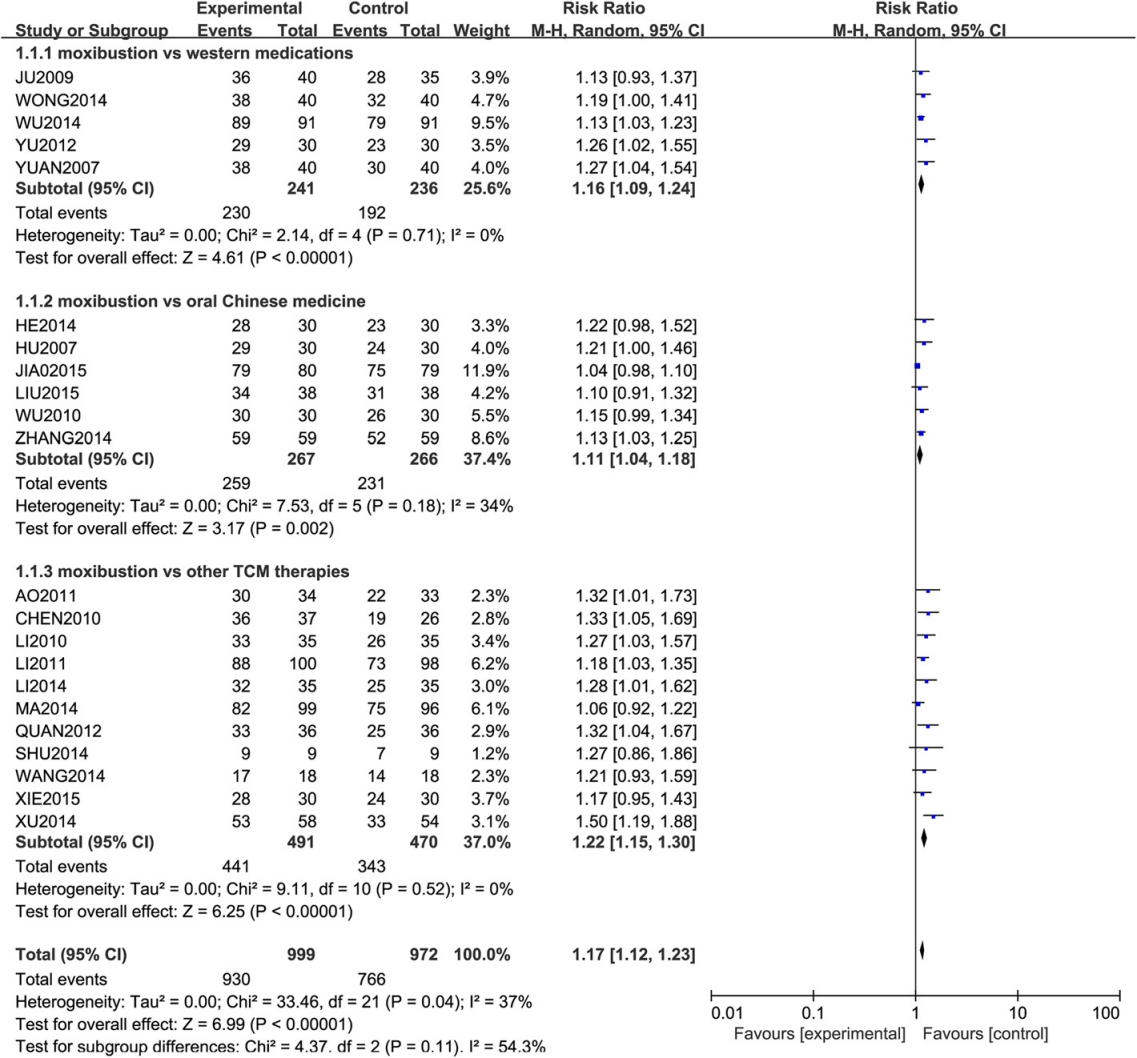
Forest plot and meta-analysis of effective rate

The overall meta-analysis (Fig. 3) demonstrated that moxibustion was more effective for insomnia than western medications, oral Chinese medicine and other TCM therapies (RR = 1.17, 95 % CI 1.12 to 1.23, *P* < 0.00001). There was an evidence of significant heterogeneity between the trials (χ^2^ = 33.46, *P* = 0.04, I^2^ = 37 %) and tests for subgroup differences showed there were some potential differences between the groups (χ^2^ = 4.37, *P* = 0.11, I^2^ = 54.3 %).

The subgroup meta-analysis (Fig. 3) demonstrated that moxibustion was more effective for insomnia than western medications [54, 59–62] (RR = 1.16, 95 % CI 1.09 to 1.24, *P* < 0.00001), oral Chinese medicine [63–68] (RR = 1.11, 95 % CI 1.04 to 1.18, *P* = 0.002), and other TCM therapies [5, 69–78] (RR = 1.22, 95 % CI 1.15 to 1.30, *P* < 0.00001). There were no evidence of significant heterogeneity between the trials comparing moxibustion vs. western medications [54, 59–62] (χ^2^ = 2.14, *P* = 0.71, I^2^ = 0 %) and moxibustion vs. other TCM therapies [5, 69–78] (χ^2^ = 9.11, *P* = 0.52, I^2^ = 0 %). But there was an evidence of significant heterogeneity between the trials comparing moxibustion vs. oral Chinese medicine [63–68] (χ^2^ = 7.53, *P* = 0.18, I^2^ = 34 %).

To account for clinical heterogeneity and subgroup differences probably arising from the use of different criteria to evaluate the effectiveness of moxibustion therapy for insomnia, a sensitivity analysis of trials using only GCTNPCM criteria was conducted. The effectiveness of moxibustion classified according to GCTNPCM criteria was described in 12 trials (moxibustion vs. western medications in 3 trials [60–62], moxibustion vs. oral Chinese medicine in 3 trials [63, 66, 67] and moxibustion vs. other TCM therapies in 6 trials [5, 70, 72, 74, 75, 77]).

**Fig. 4:**
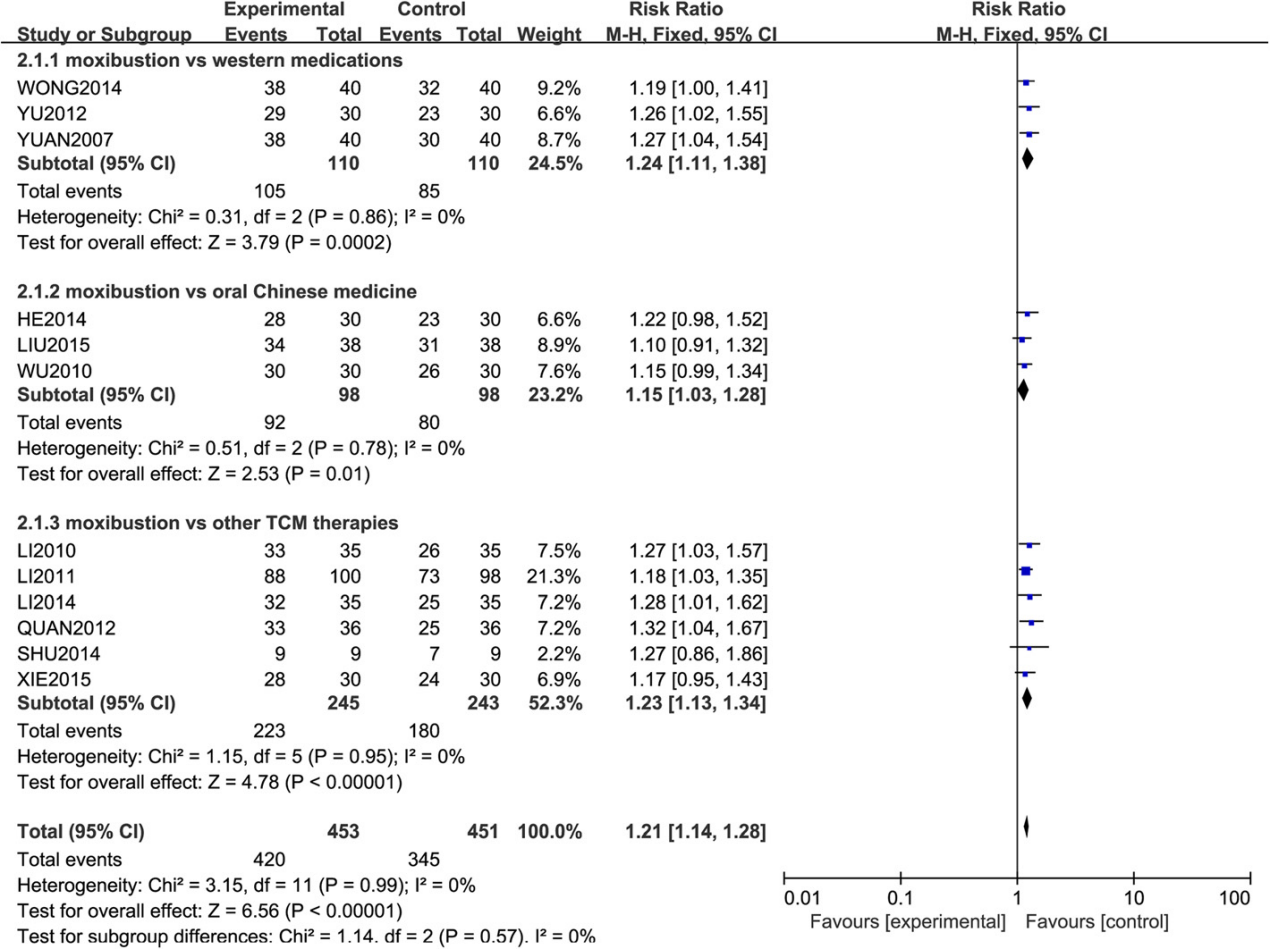
Forest plot and meta-analysis of effective rate for sensitivity

In sensitivity analysis (Fig. 4), the overall meta-analysis demonstrated that moxibustion was significantly more effective for insomnia than western medications, oral Chinese medicine and other TCM therapies (RR = 1.21, 95 % CI 1.14 to 1.28, *P* < 0.00001). There was no evidence of significant heterogeneity between the trials (χ^2^ = 3.15, *P* = 0.99, I^2^ = 0 %) and tests for subgroup differences showed there were no potential differences between the groups (χ^2^ = 1.14, *P* = 0.57, I^2^ = 0 %).

In sensitivity analysis (Fig. 4), the subgroup meta-analysis demonstrated that moxibustion was still more effective for insomnia than western medications [60–62] (RR = 1.24, 95 % CI 1.11 to 1.38, *P* = 0.0002), oral Chinese medicine [63, 66, 67] (RR = 1.15, 95 % CI 1.03 to 1.28, *P* = 0.01), and other TCM therapies [5, 70, 72, 74, 75, 77] (RR = 1.23, 95 % CI 1.13 to 1.34, *P* < 0.00001), indicating that the outcomes of the original meta-analysis are robust. There were no evidence of significant heterogeneity between the trials comparing moxibustion vs. western medications [60–62] (χ^2^ = 0.31, *P* = 0.86, I^2^ = 0 %), moxibustion vs. oral Chinese medicine [63, 66, 67] (χ^2^ = 0.51, *P* = 0.78, I^2^ = 0 %), moxibustion vs. other TCM therapies [5, 70, 72, 74, 75, 77] (χ^2^ = 1.15, *P* = 0.95, I^2^ = 0 %).

### Publication bias

**Fig. 5:**
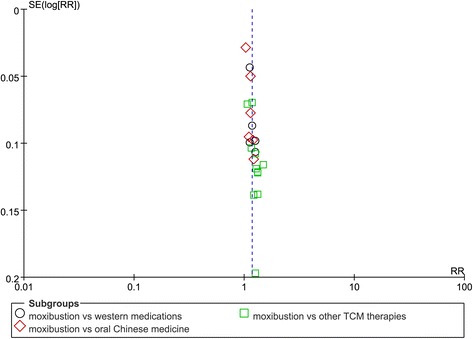
Funnel plot

There was an obvious publication bias (Fig. 5. Begg’s test Pr = 0.001).

### Adverse events

Adverse events associated with moxibustion therapy for insomnia were described in 3 studies. Wu [59] reported 3 cases of headache, 5 cases of fatigue, 1 case of constipation, and 2 cases of diarrhea in the moxibustion group, and 53 cases of bitter taste in the mouth, 6 cases of thirst, 8 cases of myasthenia, 16 cases of drowsiness, 15 cases of vomiting, and 91 cases of abstinence symptoms in the estazolam group. Wang [76] reported 1 case (5.5 %) of aching in the moxibustion plus point-application group and 1 case of aching, 1 case of nausea, and 1 case of diarrhea (total 3 cases, 16.5 %) in the point-application group (*p* < 0.05). Xu [78] reported 5 cases (8.33 %) of scalp pain in the moxibustion plus head-needle acupuncture group and 4 cases (7.47 %) of scalp pain in the head -needle acupuncture group.

## Discussion

### Summary of results

The results demonstrated that moxibustion was more effective for insomnia than western medications, oral Chinese medicine, and other TCM therapies both in the overall meta-analysis and subgroup meta-analysis. However, the efficacy of moxibustion therapy for insomnia cannot be confirmed due to a high risk of bias in the included studies and the small sample sizes. Our findings indicate no serious adverse effects associated with moxibustion therapy for insomnia and a low rate of adverse events; however, only a small number of studies reported on the safety of moxibustion.

### Quality of the evidence

The results of our study should be interpreted with caution due to the high risk of bias in the included trials. In particular, risk of bias due to the random sequence generation, allocation concealment, blinding of participants, personnel, and outcome assessments and incomplete outcome data are considerations. Firstly random sequence generation and allocation concealment are important to prevent selection bias. Trials with inadequate allocation concealment report, on average, that the intervention is 18 % more “beneficial” than in trials with adequate concealment (95 % CI 5 % to 29 %) [79]. Secondly, blinding of participants, personnel, and outcome assessments are important to prevent performance bias and detection bias. Thirdly, as insomniacs are liable to be anxious and drop out if they feel the treatment invalid, according to our clinical experience, especially in a long treatment duration, detailed information about how to prevent dropouts or whether to use ITT to deal with data of dropouts plays irreplaceable role when warranting the validity and the reliability of data. But most of the included study did not report these 4 parts clearly or rightly.

### Limitations

First, diagnostic criteria for insomnia varied between included trials. As the objective of this study was to investigate the effectiveness of moxibustion for insomnia, subgroup analyses according to diagnostic criteria were not performed. This is in accordance with other meta-analyses of TCM for insomnia [39, 80]. Second, overall risk of bias in the 22 included trials was high. Third, the included trials relied on different criteria to classify the effectiveness of moxibustion which could lead to heterogeneity between the trials or subgroup differences, and the number of studies according to different effectiveness criteria is not enough if we made subgroup. However, a sensitivity analysis focusing on GCTNPCM criterion indicated the results of the meta-analysis were robust. Fourth, there was an obvious publication bias. The reason may be some Asian countries, including China, publish an unusually high proportion of positive results [81]. Fifth only 3 studies pay attention to adverse effect.

### Implications

Our data suggest that additional high-quality trials are warranted to determine the benefits of moxibustion for insomnia. When designing and reporting future RCTs on moxibustion for insomnia, we recommend that the CONSORT 2010 statement [82, 83], which consists of a 25-item checklist to determine trial quality and rigor, should be used as a guideline. All clinical trials should be registered prior to the enrollment of the first patient, according to the International Committee of Medical Journal Editors statement [84]. Randomization methods should be clearly described and fully reported. Although blinding may be difficult, the blinding of patients and outcome assessors should be attempted. Well-defined and widely recognized diagnostic or classified criteria, such as ICD-10 or the CCMD-3, should be employed to make a precise clinical diagnosis of insomnia and increase comparability between trials. Additionally, because insomnia is a highly heterogeneous disease and presents various etiologies and severities, moxibustion is likely to differentially affect various patient subgroups. Therefore, future clinical trials should focus on particular subgroups or include a very large sample size to delineate the effects of moxibustion on different patient types. Interventions should include appropriately long treatment periods, treatment frequencies, and follow-up periods. The severity of insomnia may vary despite the presence of absence treatment; thus, a longer follow-up period with serial measurements of outcomes is important to determine the long-term effects of moxibustion. Internationally recognized and validated outcome measurements should be consolidated and used consistently. Appropriate statistical analyses should be carried out for the baseline data and ITT analysis is recommended in case of dropouts or withdrawal. Trials should have a sufficiently large sample size, ideally based on formal power calculations.

## Conclusion

It is difficult to get the conclusion regarding the effectiveness and safety of moxibustion for primary insomnia due to insufficient evidence, such as the high risk of bias in the included studies, small sample sizes, and few reports on adverse effects. Moxibustion should be considered as a novel therapeutic option for insomnia, and more rigorous clinical trials of moxibustion therapy for insomnia are needed to assess its effects.

## Abbreviations

5-HT, 5-hydroxytryptamine; CBT, cognitive behavioral therapy; CCMD-3, Chinese classification and criteria for mental disorders 3^rd^ edition; CDT&ETCMD&S, Criteria of Diagnosis and Therapeutic Effects for TCM Disease and Syndrome; CENTRAL, Cochrane Central Register of Controlled Trials; CNKI, Chinese National Knowledge Infrastructure; GCTNPCM, Guideline for Clinical Trials of New Patent Chinese medicines; ICD-10, International Classification of Disease 10^th^ Version; ITT, intention-to-treat; PSQI, Pittsburgh Sleep Quality Index; RCTs, randomized controlled trials; TCM, traditional Chinese medicine; VIP, VIP information database.
